# Antifungal activity of two oxadiazole compounds for the paracoccidioidomycosis treatment

**DOI:** 10.1371/journal.pntd.0007441

**Published:** 2019-06-04

**Authors:** Franciele Abigail Vilugron Rodrigues-Vendramini, Daniella Renata Faria, Glaucia Sayuri Arita, Isis Regina Grenier Capoci, Karina Mayumi Sakita, Silvana Martins Caparroz-Assef, Tania Cristina Alexandrino Becker, Patrícia de Souza Bonfim-Mendonça, Maria Sueli Felipe, Terezinha Inez Estivalet Svidzinski, Bernard Maigret, Érika Seki Kioshima

**Affiliations:** 1 Department of Clinical and Biomedical Analysis, State University of Maringá, Maringá, Brazil; 2 Department of Pharmacology and Therapeutics, State University of Maringá, Maringá, Brazil; 3 Department of Basic Health Sciences, State University of Maringá, Maringá, Brazil; 4 Department of Cell Biology, Institute of Biological Sciences, University of Brasília, Brazil; 5 LORIA, Lorraine University, Nancy, France; University of Tennessee, UNITED STATES

## Abstract

Paracoccidioidomycosis (PCM) is a neglected disease present in Latin America with difficulty in treatment and occurrence of serious sequelae. Thus, the development of alternative therapies is imperative. In the current work, two oxadiazole compounds (LMM5 and LMM11) presented fungicidal activity against *Paracoccidioides* spp. The minimum inhibitory and fungicidal concentration values ranged from 1 to 32 μg/mL, and a synergic effect was observed for both compounds when combined with Amphotericin B. LMM5 and LMM11 were able to reduce CFU counts (≥2 log_10_) on the 5^th^ and 7^th^ days of time-kill curve, respectively. The fungicide effect was confirmed by fluorescence microscopy (FUN-1/FUN-2). The hippocratic screening and biochemical analysis were performed in Balb/c male mice that received a high dose of each compound, and the compounds showed no *in vivo* toxicity. The treatment of experimental PCM with the new oxadiazoles led to significant reduction in CFU (≥1 log_10_). Histopathological analysis of the groups treated exhibited control of inflammation, as well as preserved lung areas. These findings suggest that LMM5 and LMM11 are promising *hits* structures, opening the door for implementing new PCM therapies.

## Author summary

Paracoccidioidomycosis (PCM) is a granulomatous fungal infection with clinically severe forms and serious pulmonary sequelae. The current limited arsenal and prolonged treatment regimen demonstrate the need for new antifungals. This study reveals two fungicidal oxadiazole compounds for PCM treatment. The *in vitro* assay showed the antifungal activity and the synergic effect with Amphotericin B. The high doses administration in mice showed absence of toxicity, which allowed to demonstrate the *in vivo* antifungal activity.

## Introduction

Paracoccidioidomycosis (PCM) is an endemic fungal disease in Latin American countries, which presents high prevalence in South America. The lung is the most affected organ, mainly during chronic form, presenting pulmonary architectural distortion, which can lead to hypoxemia and hypercapnia in 90% of patients with PCM [1]. In the last 30 years, the presence of pulmonary damage ranged from 63.8 to 100% in the patients [2, 3, 4, 5]. Furthermore, this injury remains even after the treatment and promotes pulmonary fibrosis with loss of respiratory function in 50% of patients [6, 7].

Considering this worrying scenario, the current available antifungal drugs are limited. In Brazil, only three therapeutics options are available for PCM treatment, such as polyenes, sulfanilamide and triazoles. The azoles action on the sterol biosynthetic pathway leads to many side-effects. Amphotericin B (AmB), a polyene, is the antifungal of choice in severe and acute cases. The treatment time should be as short as possible, between two and four weeks, due to its high toxicity [8]. The sulfanilamide is treatment options according to the severity of the disease; however, several disadvantages have been reported such as hypersensitivity reactions, gastrointestinal symptoms, hemolytic anemia, agranulocytopenia and crystalluria [9]. On the other hand, the most commonly used antifungal agent for treating mild and moderate forms of PCM is itraconazole (ITZ), but the time of therapy may reach 18 months and presents some collateral effects [10].

The major therapeutic challenges of this disease are the long period of continuous use of systemic antifungals, the possibility of relapses and the appearance of sequelae in the lung [1]. This, associated with the limited antifungal arsenal, evidences the necessity of the emergence of a new antifungal class.

Thus, the development of a drug that selectively acts on the target pathogenic fungi without producing collateral damage to mammalian cells is a pharmacological challenge. Biotechnological methods have become an important approach in pharmaceutical drug research and development. For example, the *in silico* methodologies not only reduce the cost associated with drug discovery, but they may also reduce the time it takes for a drug to reach the market [11]. This is a modern strategy to explore the interaction of compounds with a specific target [12].

By comparative genomics, ten potential targets for drugs occurring in eight human pathogenic fungi—*Candida albicans*, *Cryptococcus neoformans*, *Aspergillus fumigatus*, *Blastomyces dermatitidis*, *Coccidioides immitis*, *Histoplasma capsulatum*, *Paracoccidioides brasiliensis* and *Paracoccidioides lutzii*—were described [13]. One of these targets is thioredoxin reductase (Trr1), a flavoenzyme that acts primarily on resistance to oxidative stress, and it is essential to cell growth [14]. The *trr1* mutation may result in hypersensitivity to hydrogen peroxide and to high temperatures [15]. In addition, this Trr1 isoform is found only prokaryotes and fungi [14]. Therefore, Trr1 a good target for the development of new anti-PCM therapies [16]. By molecular modeling and virtual screening, several compounds were selected as Trr1 ligands. Preliminary results showed that two compounds, which belong to the oxadiazole class, present antifungal activity against important pathogenic fungi such as *Candida* spp., *Cryptococcus neoformans* and *Paracoccidioides* spp. [17]. For this purpose, the antifungal activity of two oxadiazole compounds selected by *in silico* methods was tested both *in vitro* and *in vivo* against *Paracoccidioides* spp.

## Methods

### Ethics statement

All the procedures were performed according to the regulations of the Ethical Committee for Animal Experimentation, State University of Maringá, Brazil (approval no. CEUA 9810191015, 22/04/2016). The animal’s experimentation were conducted according to the Guideline for the Care and Use of Laboratory Animals (CONCEA).

### Compounds

**Fig 1:**
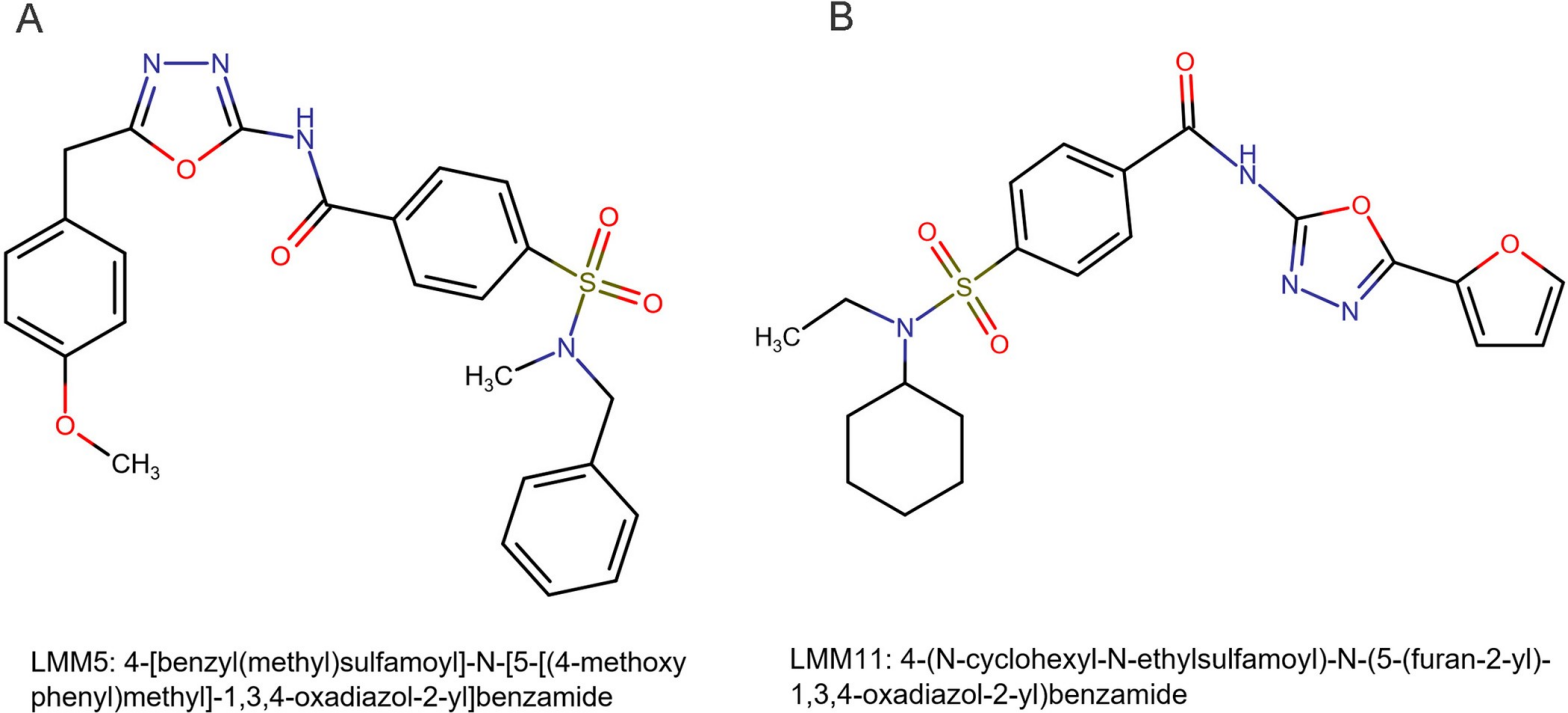
Chemical structure of two oxadiazole compounds named LMM5 (A) and LMM11 (B).

The compounds selected by virtual screening against thioredoxin reductase were commercially purchased from Life Chemicals Inc. (Burlington, ON, Canada). These compounds were named by LMM5 is 4-[benzyl(methyl)sulfamoyl]-N-[5-[(4-methoxyphenyl)methyl]-1,3,4-oxadiazol-2-yl]benzamide, and the chemical name of LMM11 is 4-[cyclohexyl(ethyl)sulfamoyl]-N-[5-(furan-2-yl)-1,3,4-oxadiazol-2-yl]benzamide (Fig 1). The stock solutions were prepared in dimethyl sulfoxide (DMSO) at concentration 100 μg/mL for LMM11 and 50 μg/mL for LMM5.

### Fungal and culture condition

Nine isolates of *Paracoccidioides* spp. were used, three *P*. *brasiliensis* (Mg0113, Mg0213 and Pb18), three *P*. *lutzii* (Pb01, 8334 and Mg0114) and three isolates not identified yet at species level (Mg0116, Mg0216, Mg0115). *Candida parapsilosis* (ATCC 22019) and *Candida krusei* (ATCC 6258) were included for quality control. The isolates are part of the collection from Laboratory of Medical Mycology of the State University of Maringá, Brazil. The yeast phase was maintained by weekly passaging at 37°C in Fava Netto's solid medium. For each experiment, the viability of *Paracoccidioides* spp. was determined by counting viable cells in a Neubauer chamber by the trypan blue method. The assays were performed with ≥80% of viable cells [18].

### Animals

Balb/c male mice, approximately six weeks old, with an average weight of 20 g, were raised at animal facilities of the State University of Maringá, Brazil. The animals were divided in groups and maintained in ventilated cages, with free access to tap water and food, in a controlled animal facility having a constant temperature of 23°C and a 12 h light/dark cycle.

### Minimum inhibitory and fungicidal concentrations assays

The minimum inhibitory concentration (MIC) was determined by the broth microdilution method, following the standard methodology by the Clinical Laboratory Standards Institute (CLSI) published in document M-27A3, with modification for *Paracoccidioides* spp. [19, 20]. The oxadiazoles compounds’ concentrations ranged from 1 to 512 μg/mL. The inoculum was adjusted to 2 × 10^4^ yeast cells/mL and diluted 1:2 into a 96-well plate with RPMI-1640 medium. Negative controls were only medium without inoculum, and positive controls were medium plus inoculum. The incubation time was 5 days at 37°C. Interpretation of the growth *cutoff* point was performed visually based on the comparison of growth in the positive control wells. The MIC values was defined as the lowest oxadiazoles concentration that resulted in at least an 80% reduction in growth relative to the positive control [21]. For AmB, it was considered to be the concentration causing 100% inhibition compared to the control without the antifungal drug. The drug controls were performed with AmB against *C*. *parapsilosis* (ATCC 22019) and *C*. *krusei* (ATCC 6258), according to the CLSI (document M27-A3) [19].

The minimum fungicidal concentration (MFC) of each compound was determined by transferring aliquots of 5 μL of each well from MIC microplates to brain-heart infusion (BHI) agar plates and incubating at 37°C for 7 days. The fungicidal activity was considered the lowest drug concentrations at which no colonies were able to grow. The following assays were performed with isolate Pb18.

### Time-kill curve assay

The *P*. *brasiliensis* isolate Pb18 was cultivated in McVeigh Morton Chemically Defined Culture Medium (MMcM) for 7–10 days at 37°C under agitation at 150 rpm, to obtain yeast cells with typical multiple budding [22]. This culture was adjusted to 2.5 × 10^4^ CFU/mL and treated with different concentrations of LMM5 and LMM11 (8 and 16 μg/mL) for 1, 3, 5, 7 and 14 days at 37°C. The untreated yeasts were used as controls. At each time interval, yeasts of each group were diluted in phosphate-buffered saline (PBS), and 100 μL was plated on Brain Heart Infusion (BHI) agar medium supplemented with 5% of Pb18 culture filtrate and 4% of Fetal Bovine Serum and incubated at 37°C for at least 14 days. The CFU were counted. The effect was considered fungicidal only when the CFU reduction was 3 log_10_ (≥99.9%); otherwise, it was considered fungistatic [23].

### LIVE/DEAD assay

The metabolic activity of yeast cells of Pb18 was analyzed after exposure to the MIC concentrations of LMM11 and LMM5 (both 8 and 16 μg/mL, each). The assay was performed using FUN-1 and FUN-2 stains according to the manufacturer's protocol (Molecular Probes). Yeasts were suspended in MOPS buffer containing 2% glucose. The fungal cell activity was estimated with 0.5 μM FUN-1 (100 mM stock solution, dissolved in DMSO) and expressed as a change in the ratio of red fluorescence (k = 575 nm) to green (k = 535 nm). The viability of fungal cells was determined from examination of at least 200 cells in a biological replicate by fluorescence microscopy. A dead control was done using 70% ethanol to kill Pb18. Metabolically active cells fluoresce as red in their structures, while dead cells or cells with little or no metabolic activity exhibit bright diffuse green cytoplasmic fluorescence and lack of intravacuolar fluorescent inclusions [24].

### Checkerboard assay

AmB was chosen to test in combinations with LMM5 and LMM11 against Pb18 isolate. The compounds (starting at 4× MIC) were distributed vertically while AmB (4× MIC) was added horizontally as described by Bagatin et al. [25]. A 2 × 10^4^/mL yeast cell suspension was added to 96-well plates and incubated at 35°C for 7 days. Inhibition was read visually and confirmed by XTT viability (492nm). The fractional inhibitory concentration (FIC) was determined by calculating ΣFIC = FIC_A_ + FIC_B_ = (Comb_AmB_/MIC_AmB_) + (Comb_LMM_/MIC_LMM_). For a strongly synergistic effect, FIC < 0.5; a synergistic effect, FIC < 1; an additive effect, FIC = 1; no effect, 1 < FIC < 2; and an antagonistic effect, FIC > 2 [26]. The Bliss-independent interactions were analyzed by Combenefit software [27].

### *In vivo* toxicity determination

Male Balb/c mice at 6 weeks old were divided into four groups: Control group treated with vehicle (PBS, DMSO 1%, and Pluronic F-127 0.2%); LMM5 group treated intraperitoneally with LMM5 at 25 mg/kg; and LMM11 group treated intraperitoneally with LMM11 at 50 mg/kg. The animals were monitored by Hippocratic screening at times 0, 15, 30, 60, 120, 240 and 480 minutes. After the 14^th^ day, the mice were anesthetized for blood collection and euthanized. The biochemical examinations were performed, and the liver, heart and kidneys were weighed, on the day of euthanasia. The assay was performed in accordance with Salci et al. [28].

### The *in vivo* antifungal activity

After inoculation with 10^6^ Pb18 yeast (intratracheally), animals were randomly divided into experimental groups: LMM5, LMM11, ITZ (group treated with itraconazole) and control. The treatment started after 24 hours of infection. The compounds and ITZ were administered at 5 mg/kg, once per day for 14 days, intraperitoneally. The animals were euthanized by isoflurane vaporizer, and the number of CFU/g of the lung tissue was determined [20].

### Histopathological analysis

Mice were euthanized 15 days post-infection, and lungs were collected. The organs were fixed in 10% formalin and embedded in paraffin. Five-micrometer sections were stained with Grocott's methenamine silver (GMS) and counterstained with hematoxylin–eosin (H&E). From the histological sections of the lung, the area was determined, and CFU/mm^2^ were counted. The calculation consists of the total number of fungal cells divided by the lung area [29]. The lung sections were analyzed about the cellular changes, and presence of fungi and inflammatory cells, using a Motic model BA310LED microscope, Moticam 5.0 MP digital camera (100, 400 and 600x magnification) and Motic software. Thus, 20 fields of at least two histological sections were classified according to the presence of inflammatory infiltrates categorized as severe (3+ or more), moderate (2+), mild (1+) and non-inflammatory (0+) [30].

### Statistical analysis

Statistical analysis of the different experimental groups was performed by GraphPad Prism software (GraphPad Software, San Diego, CA, USA). Reduction of fungal burden from *in vivo* treatment was reported as log_10_ of mean ± standard deviation using unpaired Student’s T-test. The significance of differences in histopathological score was determined by Student’s T-test. The level of significance was set as *p< 0*.*05*.

## Results

### Promising antifungal activity of new compounds

LMM5 was able to inhibit the growth of all isolates of *Paracoccidioides* spp.. 77.8% of isolates presented MIC values ranging between 8 and 32 μg/mL (Table 1). The Mg0114 isolate was the most sensitive (MIC = 1 μg/mL). All isolates presented the minimum fungicidal concentration (MFC) values similar to the MIC values. Otherwise, the MIC values of LMM11 was 8 μg/mL for most of the isolates (88.9%). The MFC were 8 and 16 μg/mL, corresponding to 66.7 and 33.3% of the isolates, respectively (Table 1). The susceptibilities of isolates to AmB are shown by MIC values of 2 μg/mL (66.7% of the isolates) and 1 μg/mL (33.3%).

**Table 1:** Minimum inhibitory and fungicidal concentrations of LMM5 and LMM11 against *Paracoccidioides* spp. isolates.

**Isolate**	**MIC AmB μg/mL**	**MIC LMM5 μg/mL**	**MFC LMM5 μg/mL**	**MIC LMM11 μg/mL**	**MFC LMM11 μg/mL**
**Pb18**	2	16	16	8	8
**Mg0115**	2	32	32	8	8
**Pb01**	1	32	32	8	16
**Mg0114**	1	1	1	8	8
**8334**	2	8	8	8	8
**Mg0113**	1	4	4	4	8
**Mg0116**	2	32	32	8	16
**Mg0216**	2	32	32	8	8
**Mg0213**	2	32	32	8	16

Abbreviations: MIC—Minimum inhibitory concentration; MFC—Minimum fungicidal concentration.

### Fungicide kinetic of the new compounds against *P*. *brasiliensis*

**Fig 2:**
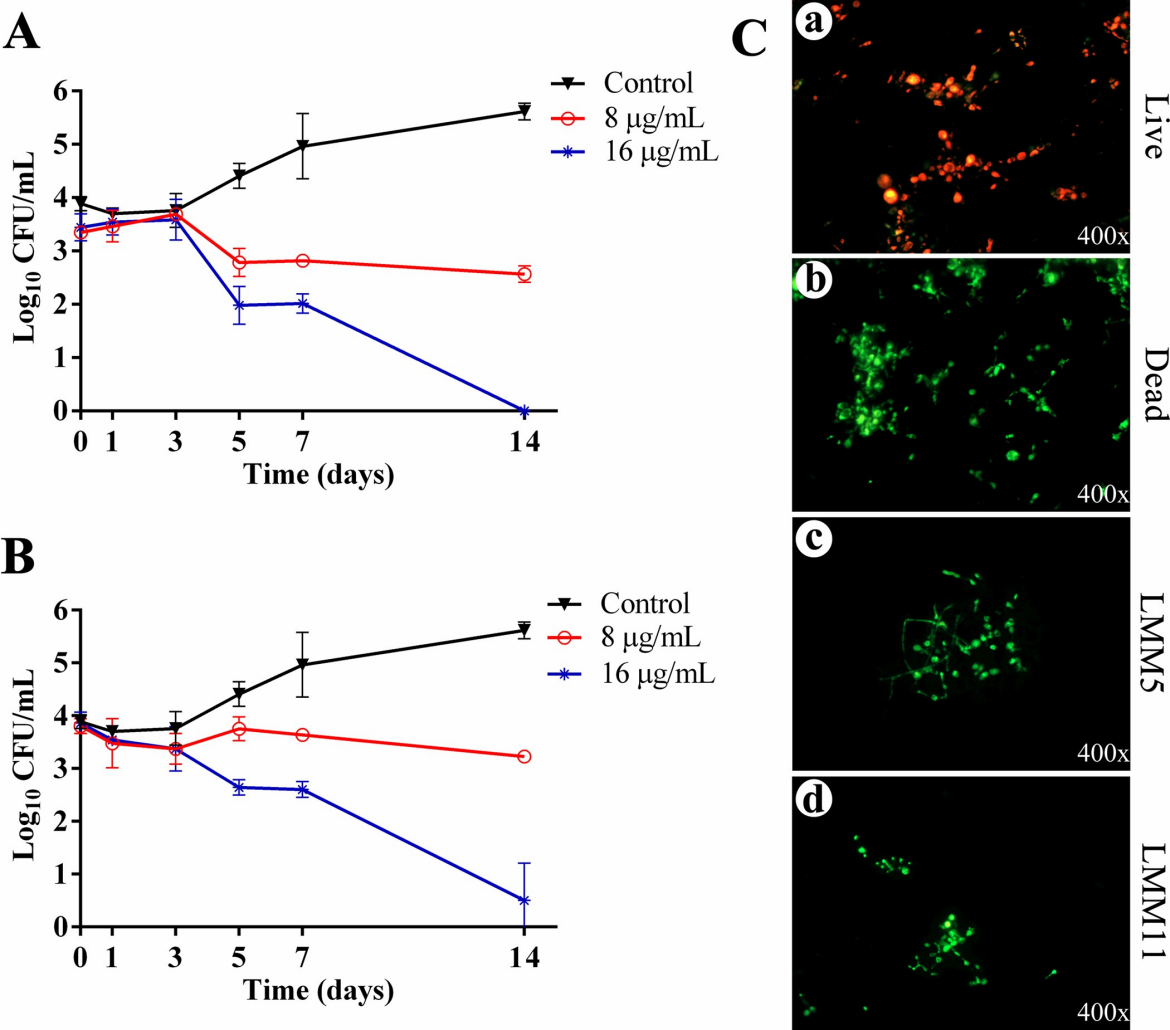
Fungicidal activity of LMM5 and LMM11. Time-kill curves of Pb18 treated with compound LMM5 **(A)** and LMM11 **(B)** were performed in two concentrations each—8 μg/mL and 16 μg/mL—at 37°C for 14 days. **(C)** Photomicrographs of *P*. *brasiliensis*, stained with FUN-1 and FUN-2, were obtained by fluorescence microscopy with 400x magnification. (a) Positive control—high metabolic activity of live fungal cells with yellow-orange fluorescence. (b) Negative control—dead cells with diffuse bright green fluorescence. (c) Fungal cells show diffuse green fluorescence after 7 days of interaction with LMM5 (16 μg/mL). (d) Pb18 cells exhibiting diffuse bright green fluorescence after 7 days of contact with LMM11 (16 μg/mL).

The change in growth over time was evaluated by time-kill curves during 14 days (Fig 2). The yeast treated with LMM5 or LMM11 exhibited 80% reduction in the Pb18 cell viability from the 5^th^ day post-treatment. The fungicidal profile was determined by CFU reductions of ≥3 _log10_ as compared with control growth. The LMM5 fungicidal profile can be observed from the 7^th^ day (Fig 2A). For LMM11, the fungicidal effect was detected on the 7^th^ day (16 μg/mL) as shown in Fig 2B. In addition, the largest difference between groups was observed on the 14^th^ day, for both compounds.

The time-kill curve results were corroborated by LIVE/DEAD assay, in which the cellular viability was evaluated by fluorescence microscopy. For this evaluation, Pb18 cells were treated with LMM5 (Fig 2C) or LMM11 (Fig 2D). Both compounds were able to produce a diffuse bright green fluorescence profile indicating cell death or yeast with little metabolic activity. This fluorescence profile is quite different from what was observed in the control group (not treated), in which live cells presented yellow-orange intravacuolar structures.

### Synergistic fungicidal effect

**Fig 3:**
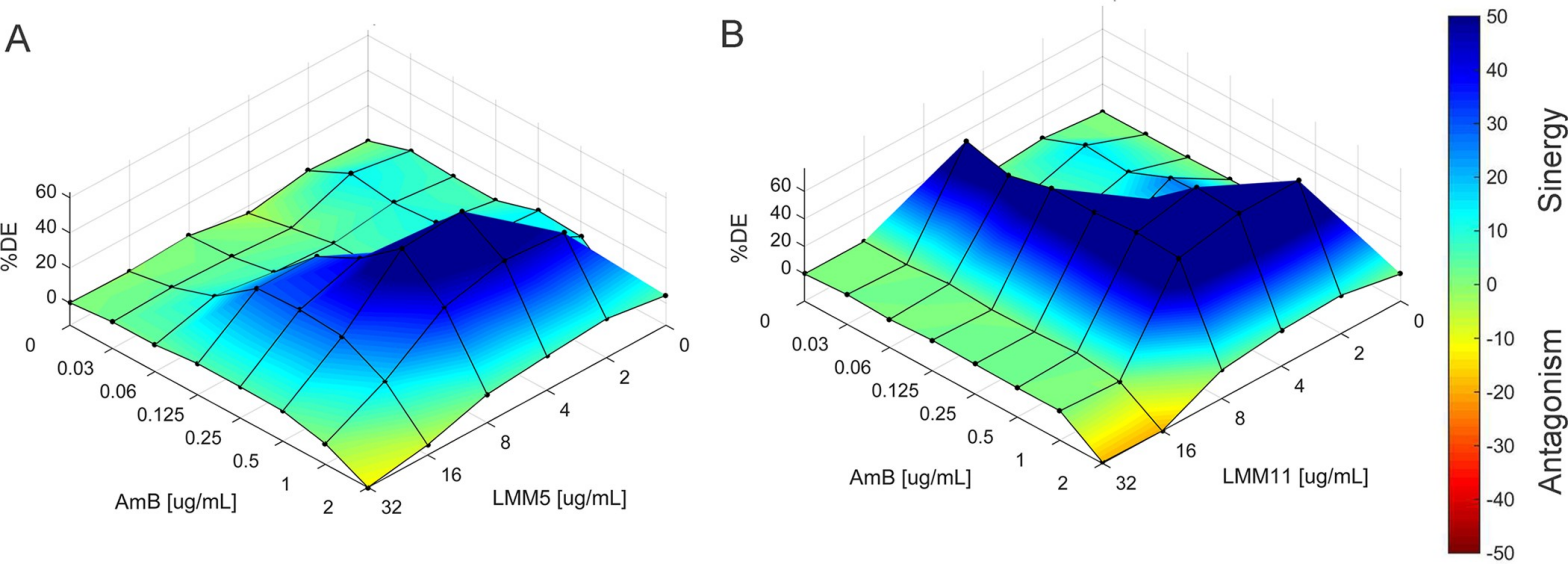
The Bliss independence surface analysis for *in vitro* combinations of Amphotericin B and oxadiazole compounds against *P*. *brasiliensis*. **(A)** Compound LMM5 **(B)** Compound LMM11. The *x* and *y* axes represent the efficacies of AmB and oxadiazole, respectively. The *z* axis is the percent DE (%DE). The zero plane represents Bliss-independent interactions, whereas the volumes above the zero plane represent statistically significant synergistic (positive DE) interactions. The magnitude of interactions is directly related to DE.

AmB, when combined with LMM5 or LMM11, showed better antifungal activity than alone, reducing the MIC value from 2 to 0.5 μg/mL. The new compounds’ interactions with AmB reduced the three-fold MIC value of LMM5 (from 32 to 4 μg/mL) and the two-fold MIC value of LMM11 (from 16 to 4 μg/mL). These results indicate a synergistic effect of AmB with LMM5 or LMM11 (Table 2). The synergic effect revealed by FIC values was validated by the result of the Bliss independence surface analysis. In this way, the AmB combination with each of the oxadiazoles showed predominance of blue areas, indicating a positive ΔE and thus confirming the synergic capacity (Fig 3).

**Table 2:** The effect of the combinations of AmB and LMM5 or LMM11 against *P*. *brasiliensis* (Pb18).

Compound	MIC (μg/mL)			FIC model	
	Alone	Combination	0030	∑ FIC	Interpretation
**LMM5**	32	4	0.125	0.375	Strong synergistic
**AmB**	2	0.5	0.25		
**LMM11**	16	4	0.25	0.5	Synergistic
**AmB**	2	0.5	0.25		

Abbreviations: MIC—Minimum inhibitory concentration; AmB—Amphotericin B; FIC—Fractional inhibitory concentration; ∑ FIC—Summative fractional inhibitory concentration.

### *In vivo* toxicity assay for LMM5 and LMM11

**Fig 4:**
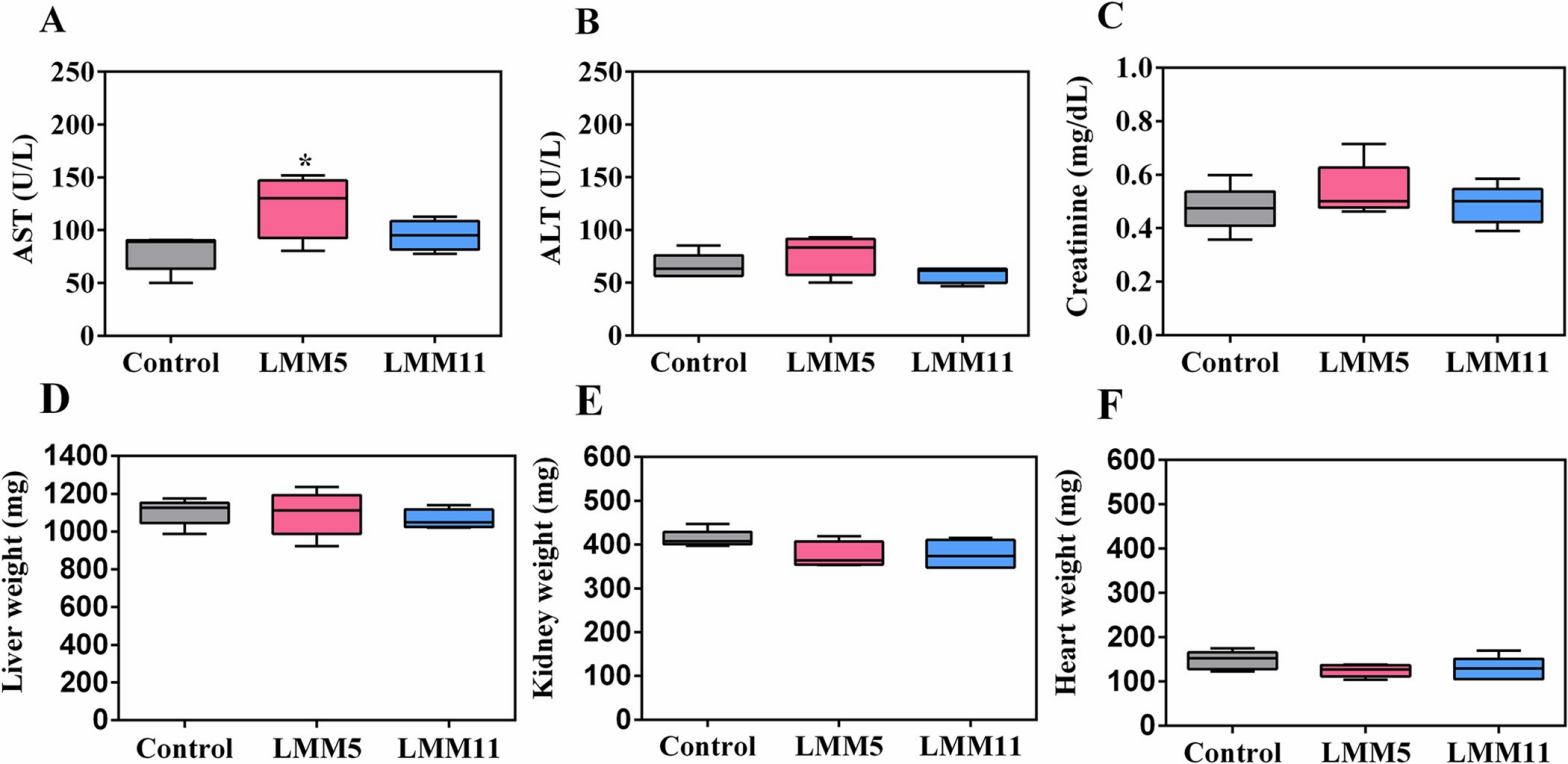
Biochemical parameters and organ weights of mice exposed to high concentrations of LMM5 and LMM11. **(A)** Serum levels of amino transferase aspartate (AST). **(B)** Serum levels of amino transferase alanine (ALT). **(C)** Serum levels of creatinine. **(D)** Liver weight of mice after 14^th^ day of the single dose administration. **(E)** Kidney weight of mice after 14^th^ day of the single dose administration. **(F)** Heart weight of mice after 14^th^ day of the single dose administration. The control group received the vehicle used for administering the compounds (PBS, 1% DMSO and 0.2% Pluronic). LMM5 group was administered with 25 mg/kg. LMM11 group was administered with 50 mg/kg. The control, LMM5 and LMM11 were administered in a single dose. * represents a significant difference in relation to the control group *(p <0*.*05*).

*In vivo* toxicity parameters showed mild behavioral changes, such as abdominal contortion and motor impairment, within 30 minutes after intraperitoneal administration of the compounds in all groups evaluated. After this period, no alterations were observed. For both compounds, it is important to note that there were no differences in the body weight of animals, in the hematological profile and in the macroscopic analysis of the organs after 14 days. Regarding the biochemical parameters, although the serum levels of amino transferase aspartate (AST) from mice receiving LMM5 were significantly higher than that from the control (*p <0*.*05*), their values were within those expected for normal mice (Table 3). Similarly, the LMM11 group showed no statistical differences in the AST values compared to that from the control group (Fig 4A). Both amino transferase alanine (ALT) and creatinine levels did not present a statistical difference between the groups evaluated (Fig 4B and 4C). According to Fig 4D, the liver did not exhibit changes in its weight, while in the kidney, although there was a slight reduction in the kidney weight of animals receiving LMM5 and LMM11, no statistical difference was found (*p >0*.*05*) (Fig 4E). There were no significant changes in heart weight (Fig 4F).

**Table 3:** Serum levels of aminotransferase aspartate (AST), aminotransferase alanine (ALT) and creatinine evaluated in male Balb/c mice after application of a single dose of LMM5 and LMM11.

	AST (U/L)	ALT (U/L)	Creatinine (mg/dL)
**Control**	88.7 ± 17.3	65.7 ± 11.7	0.4 ± 0.08
**LMM5**	130.4 ± 30.3	77.5 ± 19.1	0.5 ± 0.1
**LMM11**	95.0 ± 14.3	58.3 ± 7.9	0.5 ± 0.07
Reference values [31]	64.0–258.0	75.0–193.0	0.2–0.6

### Comparative treatment of experimental PCM

**Fig 5:**
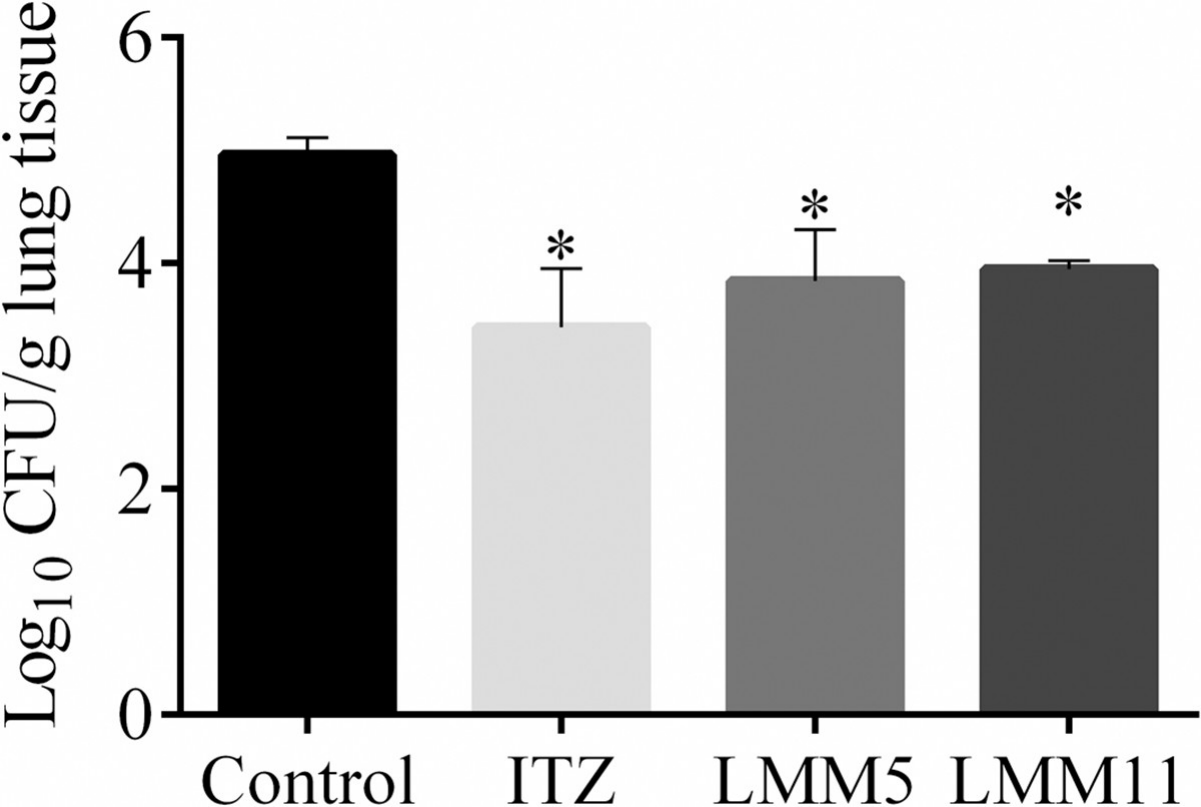
LMM5 and LMM11 therapy from PCM in murine model. The pulmonary fungal burden of mice infected by *Paracoccidioides brasiliensis* (10^6^ cells). Control: animals treated with vehicle (PBS, DMSO 1% and Pluronic 0.2%); ITZ: animals treated with itraconazole (5 mg/kg); LMM5: animals treated with compound LMM5 (5 mg/kg); LMM11: animals treated with LMM11 (5 mg/kg). All treatments were carried out for 14 days. The experiments were performed in triplicate, and the bars indicate the standard deviation. *p<0.*05*, a statistically significant reduction of colony forming units per gram of lung compared to the control.

Since LMM5 and LMM11 presented promising *in vitro* antifungal activity and no toxicity *in vivo*, the next step was to evaluate them through an *in vivo* experimental PCM treatment. Daily therapy with the new compounds for 14 days showed a significant reduction of pulmonary fungal burden in relation to the control (*p <0*.*05*) for LMM5 (1.2 Log_10_ CFU/g) and LMM11 (1.0 Log_10_ CFU/g), as well as for the group treated with ITZ (1.5 Log_10_ CFU/g) as shown in Fig 5. There was no statistical difference among the groups treated (LMM5, LMM11 and ITZ); all were equally efficient in reducing fungal burden in mice (*p>0*.*05*).

### Histopathological analysis

**Fig 6:**
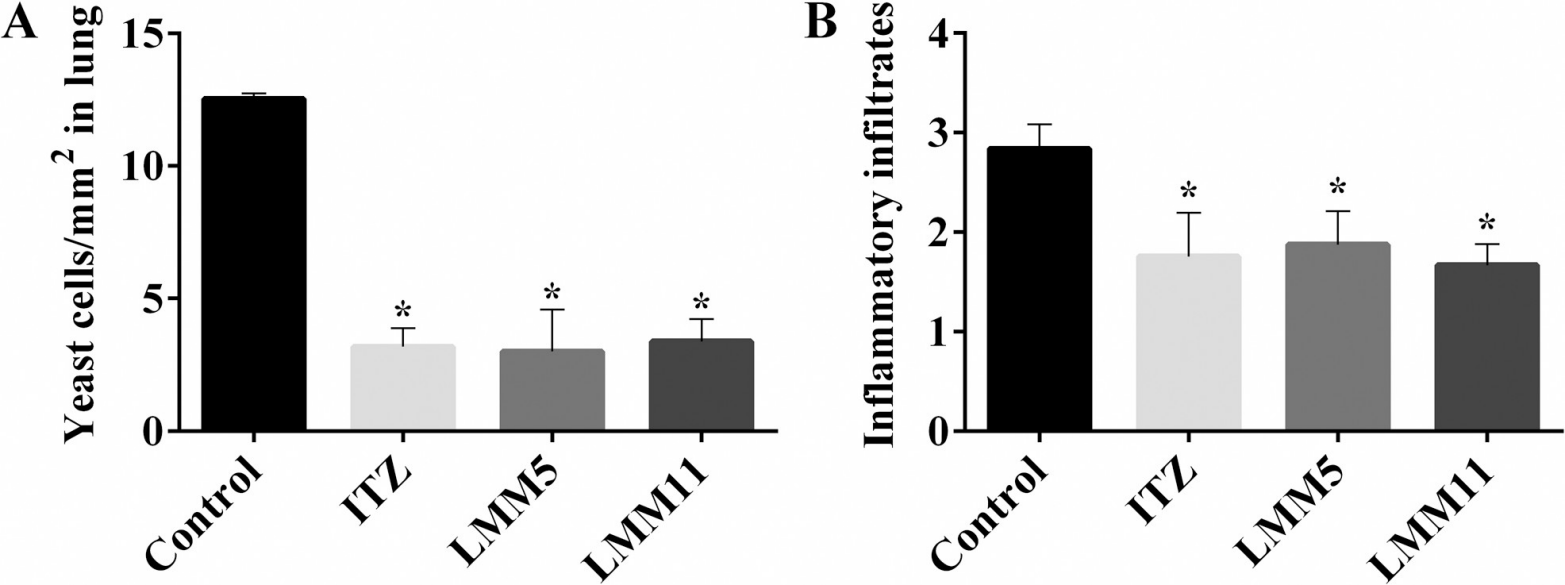
Analysis of pulmonary histological sections of the PCM experimental treatment. (A) Determination of fungal cell number per mm^2^ from histological lung sections. (B) Histological score of the total inflammatory infiltrates in the lung tissue.

Because pulmonary fibrosis is the main sequelae of PCM, even after treatment it is essential to evaluate the therapy effect of the new compounds on the inflammatory response triggered by *P*. *brasiliensis*. A quantitative analysis of the histological sections allows determination of the number of fungal cells present in each histological lung section. Fig 6A demonstrates that conventional antifungal treatment with ITZ was as effective as the new compounds in reducing the number of yeast cells/mm^2^ when compared to a control (*p<0*.*05*), but no statistical difference between compounds and ITZ was found (*p>0*.*05*). These results corroborate the significant reduction of fungal burden presented previously (Fig 5). The inflammation level, indicated by the presence of inflammatory infiltrates in lung tissue, was significantly reduced in the three treatments tested, in relation to the control (*p<0*.*05*) (Fig 6B).

**Fig 7:**
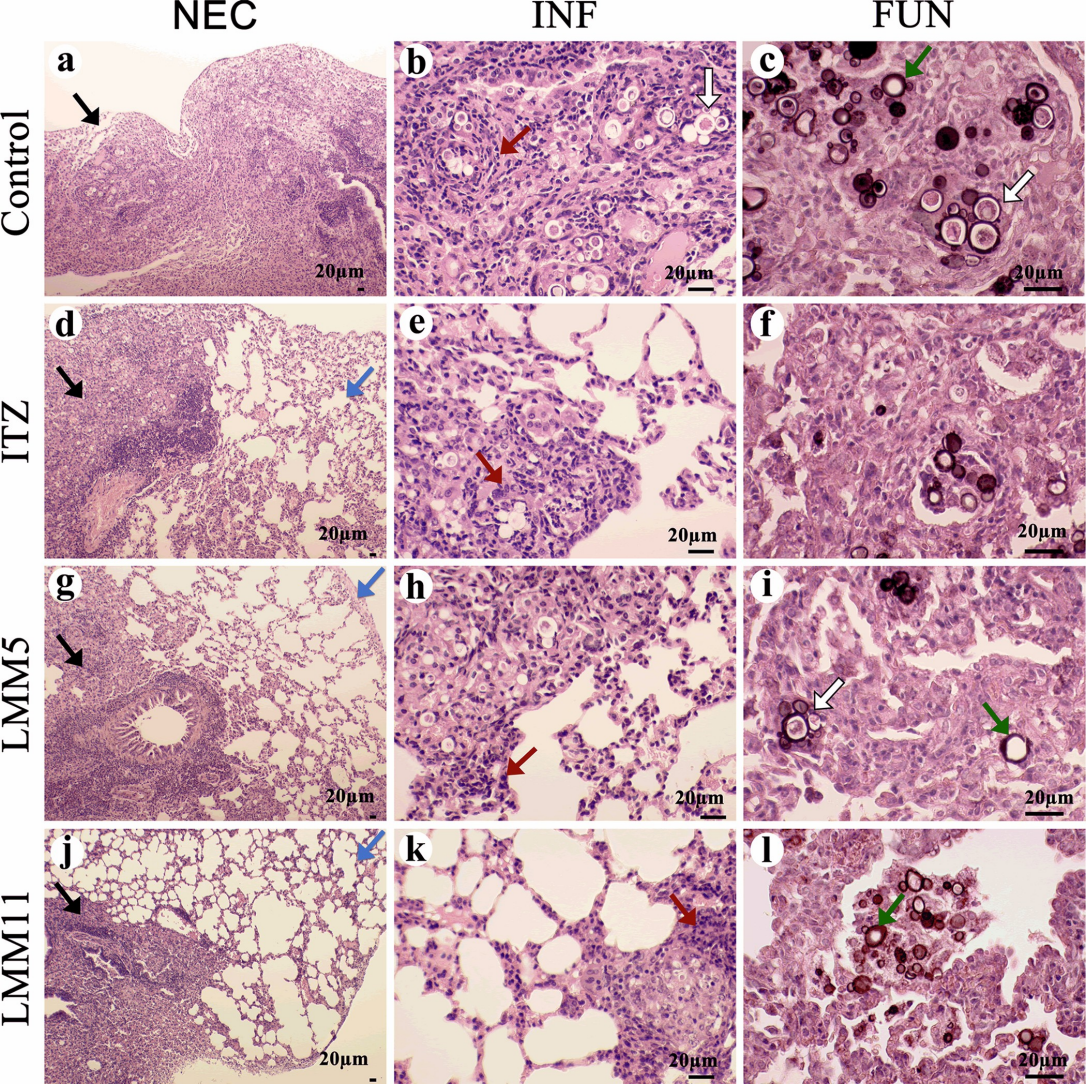
Pulmonary histopathological analysis of mice from experimental PCM treatment. Photomicrographs of lung damage, tissue sections stained by H&E (**a, b, d, e, g, h, j, k**) and H&E plus GMS (**c, f, i, l**). (**a, b, c**) Representative lung sections from mice infected and only treated with vehicle (DMSO 1% and Pluronic 0.2%) (Control); **(d, e, f**) Infected mice treated with ITZ at 5mg/kg; (**g, h, i**) Infected mice treated with LMM5 at 5mg/kg; (**j, k, l**) Infected mice treated with LMM11 at 5mg/kg. Black arrows indicate necrosis area; blue arrows point to areas of lung tissue preservation; red arrows show inflammatory infiltrate; white arrows highlight the presence of the fungus cells with viable protoplasm in the lung; green arrows indicate non-viable fungal cells stained with GSM. (**NEC**) necrosis, (**INF**) inflammatory infiltrates, (**FUN**) Fungal cells.

A qualitative analysis of the histological sections of groups treated was performed. The lungs of the infected mice that received only vehicle (DMSO 1% and Pluronic 0.2%) showed a predominance of necrotic areas, indicated by black arrows (Fig 7A). In contrast, animals treated with ITZ, LMM5 or LMM11 revealed large areas of preserved lung tissue, indicated by the blue arrows in Fig 7D, 7G and 7J, respectively. In the necrotic areas, it was possible to observe a total loss of pulmonary architecture, leading to no alveolar wall visualization (black arrows).

Severe lesions, characterized by the presence of diffuse inflammatory exudate, were also observed. An intense recruitment of mononuclear cells was detected in the control group, as indicated by red arrows (Fig 7B). In the treated groups, the presence of inflammatory infiltrates was lower (Fig 7B, 7C and 7I). The histopathological evidence showed rounded and multi-budding fungal cells presenting viable protoplasm (white arrows) and nonviable protoplasm (green arrows) in all groups analyzed (Fig 7C, 7I and 7L). Therefore, these results demonstrated that treatment with both compounds can be associated with infection control and maintenance of pulmonary architecture.

## Discussion

The key to a good prognosis is immediate treatment [32]. However, the limited number of antifungal drug classes, the need for long-term treatment, and the high toxicity and adverse effects of the drugs reduce adherence to the treatment by patients [33]. All these concerns indicate a major gap that must be filled in antifungal therapy, especially for severe mycoses treatment. Undoubtedly, the pace of discovery of new antifungals is far from reaching the current needs. However, some groups have sought new therapeutic options through *in silico* approaches.

Virtual screening based on the ligand or structure target is being performed in the identification of new compounds for PCM treatment [20, 29, 34]. The thioredoxin reductase (Trr1) has been shown as an important target for the development of molecules with antifungal activity [16, 35]. This protein is a flavoenzyme that catalyzes the reduction of NADPH-dependent thioredoxin, protecting cells against oxidative stress [36]. Thus, Trr1 comprises the three main parameters to be a potent candidate for antifungal development: it is an essential gene for fungus survival, it is absent in humans, and it is conserved in several pathogenic fungi. Therefore, it will possibly allow a broad spectrum of action [16].

LMM5 and LMM11 were selected by virtual screening as potential inhibitors of Trr1. Initial tests showed *in vitro* activity against various invasive fungal infections (IFIs) and absence of toxicity [17]. This work reports the antifungal effects of these two oxadiazoles against *Paracoccidioides* spp., presenting an inhibition profile with MIC values between 1 and 32 μg/mL. Several studies have used the *in silico* approach to identify compounds for PCM treatment [16, 20, 25, 30, 35, 37]. The MIC values for these compounds was always similar to oxadiazoles. Three inhibitors of thioredoxin reductase showed MIC values ranging from 8 to 32 μg/mL [16]. An inhibitor of chorismate synthase presented antifungal activity with MIC values between 2 and 32 μg/mL [20].

Furthermore, two homoserine dehydrogenase inhibitors demonstrated antifungal activity (MIC values 32–64 μg/mL) [25]. This group also synthetized 4-methoxy-naphthalene derivatives with antifungal activity against *Paracoccidioides* spp. (MIC values 8–32 μg/mL) [38]. A thiosemicarbazone derivative tested against 14 isolates of *Paracoccidioides* spp. showed MIC values between 3.90 and 62.50 μg/mL [39]. In addition, new chalcone derivatives presented antifungal activity with MIC values between 2.9 and 42.2 μM [34].

Drugs with fungicidal profiles are more promising than fungistatic [40]. Thus, our findings indicate that the two oxadiazoles compounds analyzed in this study are promising, because both presented fungicidal profile, especially after 7^th^ day. Fluorescence microscopy results confirmed this fungicidal profile, resulting in cell death and not only growth inhibition. A thiosemicarbazone derivative of lapachol was tested against isolate Pb18 by de Sa et al. [39], and it showed a reduction of 90% in fungal growth after the 5^th^ day; no synergistic effect with conventional drugs was detected.

The antifungal effect of this commercially available drug combined with the novel compounds was evaluated. The synergistic interaction between candidate compounds and conventional antifungal agents may reduce the need for high doses, minimizing adverse effects and providing beneficial attributes for new therapeutic strategies against PCM [41]. In this way, LMM5 exhibited a strongly synergistic effect with the most potent antifungal for PCM, and LMM11 also interacted synergistically. It suggests a possible interaction pathway with AmB, increasing its fungicidal effect [42]. It is possible to suppose that the pores opened by AmB could facilitate the access of the new compounds to the intracellular target, the thioredoxin system, leading to the synergistic interaction observed. Although chalcone derivatives have low MIC values for several isolates of *Paracoccidioides* spp., no synergistic effect was observed with AmB or another antifungal that was tested [40].

Whereas AmB is the choice drug in the most severe PCM cases, nephrotoxicity affects more than 80% of patients [43]. Recent findings reveal that hepatobiliary changes during treatment with ITZ in patients with PCM are irreversible even if they are not as frequent compared to AmB [44]. The biochemical parameters of *in vivo* toxicity assays for the new oxadiazoles were analyzed based on the references values for male Balb/c mice suggested by Araujo and collaborators [31]. Therefore, the AST, ALT and creatinine values of mice treated with high doses of oxadiazoles are within normality patterns. These findings reveal that these compounds do not present nephrotoxicity or hepatotoxicity in the murine model.

An important validation of anti-*Paracoccidioides* activity is to extrapolate to *in vivo* analysis. The experimental PCM model showed the ability of the oxadiazoles to reduce the fungal lung burden of infected mice. It is suggested that the intraperitoneal treatment with LMM5 and LMM11 reached the lungs and controlled the fungal burden as well as for itraconazole. Comparable results were found for chalcone derivatives in treatment of the PCM experimental. In which the fungal reduction was similar to itraconazole treatment [45]. Cyclopalladated treatment also demonstrated fungal burden reduction and decrease of the damages caused by infection [46].

The major challenge for patients undergoing PCM treatment is sequelae triggered by aggressive pulmonary inflammatory response, which may lead to loss of function [1]. This work evaluated how much of the lung was preserved with the different treatments. Representative images of the lung histopathology (Fig 7) demonstrated untreated animals with large areas of necrosis, filled with Pb18 yeast cells throughout the tissue. Thus, the ability of LMM5 and LMM11 to reduce fungal burden and inflammatory response in the lungs of mice infected with *P*. *brasiliensis* seems to be very promising for controlling pulmonary sequelae in PCM.

In conclusion, we have successfully demonstrated that two new oxadiazoles selected by virtual screening presented promising antifungal activity against *Paracoccidioides* spp., opening perspectives for implementing alternative PCM therapy strategies. Both *in vitro* and *in vivo* results indicated that LMM5 and LMM11 could be used as lead structures to new antifungal compounds, with fungicidal profiles and leading to reduced tissue damage caused by fungal infection.
